# The Panacea

**Published:** 1876-05

**Authors:** 


### THE PANACEA.

The great cure-all, the catholicon for the removal of untold human ills, physical and mental, which will make of life a summer sky, which will replace the darkest clouds with the gladdest sunshine, which will put a budding rose where erst flourished the ragged thorn, is the blessed habit of an implicit reliance on the wisdom and the love of Providence in every occurrence of life; of humble gratitude if it is gladsome; of uncomplaining resignation if it is adverse; abiding in the firm faith that if it is dark to-day, it will be bright to-morrow, saying and feeling of every dispensation: "Not as I will, but as Thou wilt." This is the balm of Gilead; this is perennial health; it is happiness, it is bliss.

